# Effects of tape and Kinesiotape on ankle range of motion, Single Leg Drop Jump and balance after running-induced fatigue: a cross-over clinical trial

**DOI:** 10.1371/journal.pone.0320152

**Published:** 2025-04-21

**Authors:** María García-Arrabé, Federico Salniccia, Guillermo García-Pérez-de-Sevilla, Ángel González-de-la-Flor

**Affiliations:** Department of Physiotherapy, Faculty of Medicine, Health and Sports, European University of Madrid, Spain; Lokman Hekim University, TÜRKIYE

## Abstract

The prevention and management of injuries in runners is a key area of research in sports medicine. Fatigue during running can lead to biomechanical imbalances and inhibition of stabilizing muscles, increasing the risk of injury. With the ankle being the most commonly injured joint in runners, rigid tape (RT) and Kinesiotape (KT) have been proposed as effective methods to improve joint stability and reduce injury risk. The objective of this study was to compare the effects of a KT and RT and no tape (control group) on lower limb balance, ankle dorsiflexion ROM, and electromyographic (EMG) activation of the pronator and supinator muscles of the ankle during a Single Leg Drop Jump (SLDJ) following a treadmill fatigue protocol. From March 1^st^ until April 10^th^, 2024, a cross-over clinical trial with three conditions: control group, with a RT, and with KT was conducted with n = 22 well-trained runners aged 29.29 ±  10.98 years. Lower limb stability, ankle dorsiflexion mobility, SLDJ parameters, and electromyographic activation of the ankle muscles (tibialis anterior (TA), extensor digitorum longus (EDL), peroneus longus (PL), and medial gastrocnemius (MG)) during a SLDJ were analyzed Pre and Post fatigue protocol, involving a 30-minute run at 85% of the maximal aerobic speed on a treadmill. Statistical analysis was conducted using repeated-measures ANOVA with Bonferroni correction. The RT bandage decreased ankle dorsiflexion ROM compared to both KT bandage and a control group during pre fatigue treadmill protocol measurements in the lunge and Y Balance Tests (specifically in the anterior direction). Group-by-time interaction showed significant differences for the lunge test (p =  0.045), SLDJ height (p =  0.014), flight time (p =  0.019) and ground contact time (p =  0.035). With the RT condition, the runners exhibited higher peak activation of the EDL muscle compared to the KT and control group during initial landing (p =  0.028), with a lesser decay in activation during take-off (p =  0.016). The KT showed a significant increase in the activation of the PL muscle during the first contact phase of the SLDJ (p =  0.046). Concerning flight and contact time during the SLDJ, both KT and RT proved beneficial in mitigating fatigue symptoms before and after the treadmill protocol. Regarding the RT bandage’s specific effects on muscle activation, our findings indicate that the RT group exhibited higher peak activation of the EDL muscle compared to the KT and control groups during initial landing, with a lesser decay in activation during take-off. The KT showed a significant increase in the activation of the PL muscle during the first contact phase of the SLDJ. To conclude, our study highlights the potential benefits of both KT and RT in reducing fatigue symptoms during SLDJ. The study was registered with the Australian New Zealand Clinical Trials Registry (ACTRN12624000099527) on February 2^nd^, 2024 (https://anzctr.org.au/).

## Introduction

Running is one of the most popular forms of physical activity worldwide, but it is also associated with a high prevalence of injuries [1]. Running-related musculoskeletal injuries are common among runners, with reported injury incidence rates averaging 40.2% and prevalence rates at 44.6% [1]. The most frequently affected areas include the ankle, knee, and lower leg, with ankle sprains were in the top 5 most common injuries in the incidence data [2]. Such injuries, often resulting from overuse, can compromise long-term joint stability and athletic performance. To address this issue, preventive strategies such as rigid tape(RT), Kinesiotape (KT) have gained attention for their potential to improve joint stability and decrease the likelihood of injury during physical activity.

The prevention and management of injuries in runners constitutes a constantly evolving area of research in the field of sports and medicine. During running, fatigue can induce biomechanical imbalances [3], which can inhibit stabilizing muscles [4], thus increasing the risk of injury. Ankle injuries are among the most frequent musculoskeletal injuries in runners, with ankle sprains accounting for approximately 5.8% of all running-related injuries [5]. In ultramarathoners, the prevalence of ankle injuries is even higher, with the ankle being affected in 34.5% of cases [6]. In response to this problem, RT and KT have emerged as promising approaches to improve stability [7] and prevent injuries during physical activity [8].

Ankle stability is a determining factor for optimal running performance and reducing injury risk [9]. Muscle fatigue during physical activity can compromise the capacity of the ankle stabilizing muscles, thus increasing vulnerability to injury [10,11]. To reduce this fatigue, proprioception [12] and strength training [13,14] have been shown to be beneficial in the medium term. Seeking an immediate effect, some bandages made with KT or RT seem to provide stability to the ankle joint effectively, although there is some inconsistency in the different studies [15–19].

Additionally, fatigue during running can lead to ankle instability. For this reason, some studies [9,20] propose prophylactic bandages, which are protective tapes applied preventively to stabilize joints and ligaments, thereby reducing the risk of excessive mobility that could cause sprains [21]. However, caution must be exercised when applying a RT bandage, as it can excessively restrict mobility in the ankle. This decreased range of motion (ROM) can increase the risk of ligamentous [22] and tendon [23] injuries in neighbor joints, in addition to causing pain and compromising function during physical activity [24]. However, it has also been studied that these bandages can lose their effectiveness throughout physical activity or competition [25,26]. In contrast, KT provides a more elastic and flexible bandage that offers support while maintaining mobility, potentially reducing discomfort and injury to surrounding joints [27], and KT is favorable in the recovery of muscle soreness after delayed onset muscle soreness [28].

Although KT is thought to improve proprioception without the restrictive effects of rigid taping, the evidence to support these claims is limited [29,30]. For this reason, our study aims to directly compare the effects of KT and RT, providing more clarity on their efficacy and potential benefits in reducing fatigue-related symptoms.

Muscle activation is significantly influenced by fatigue, which plays a critical role in both injury prevention and athletic performance. In amateur runners, fatigue can alter the activation patterns and compensation strategies of key muscles, such as the pronator and supinator muscles of the ankle, particularly during different phases of running. Understanding how fatigue affects these muscles, and the potential impact of different bandaging techniques on their activation, is essential for making informed decisions regarding the use of prophylactic bandages [31].

Although numerous studies have explored the effects of RT bandages and KT on ankle mobility and stability, here remains a significant lack of data regarding their influence on the activation of pronator and supinator muscles during dynamic movements such as jump landings [15,20]. This gap in the literature is especially relevant for trail runners, who frequently encounter unstable terrain and variable slopes that demand precise neuromuscular control to prevent injuries.

Additionally, the SLDJ test, a technical gesture simulating the landing phase of running, provides a relevant model to evaluate muscle activation and joint stability under conditions of induced fatigue. Given that the SLDJ involves both monopodal jump execution and landing, it mirrors the biomechanical demands of running, making it an appropriate test for assessing the effects of different bandaging techniques on muscle function and overall performance.

This study differs from previous research by focusing specifically on the electromyographic (EMG) activation of the ankle’s pronator and supinator muscles in a dynamic loading context under fatigue, an approach critical for optimizing injury prevention strategies and enhancing athletic performance in high-demand environments. Therefore, the aim of this study was to compare the effects of KT and RT on lower limb balance, ankle dorsiflexion ROM, and EMG activation of ankle stabilizing muscles during the SLDJ, following a treadmill-induced fatigue protocol. The hypothesis of our study was that KT would result in greater muscle activation and joint stability than RT under fatigued conditions, potentially offering greater support for neuromuscular control during dynamic activities.

## Materials and methods

### Study design and ethical considerations

A cross-over, longitudinal, clinical trial with three conditions (no tape, KT and RT) was conducted following the CONSORT guidelines in the Sports Performance Laboratory of the European University of Madrid (Spain), from March 1^st^ until April 10^th^, 2024. Each participant underwent all three conditions, with the order of interventions randomized to control for learning effects and bias. The variables were measured at two different time points: pre and post-fatigue protocol.

This longitudinal design allowed for the observation of pre and post fatigue treadmill protocol effects within the same participants, increasing the reliability of comparisons across the three interventions.

The investigators adhered to the Helsinki Declaration, and all participants were informed prior to their inclusion in the study. Each participant completed and signed a written consent form before being enrolled in the study. While the risks in this study are minimal, due to the potential for participants to experience discomfort or fatigue, appropriate measures to mitigate these were taken, such as offering water and reminding participants that they could leave at any time without penalty. The Ethics Research Committee of the European University of Madrid (internal code 2023-282) approved the study. Additionally, this trial was registered with the Australian New Zealand Clinical Trials Registry (ACTRN12624000099527) on February 2^nd^, 2024 (https://anzctr.org.au/).

### Sample size calculation

The sample size was calculated using the G * Power 3.1.9.2 software (G * Power©, University of Dusseldorf, Germany). This analysis employed a two-tailed hypothesis, an alpha error probability of 0.05, a beta error of 0.2, a correlation among repeated measures of 0.5 for 3 conditions and 2 times of measurement. A pilot study involving 10 participants found a partial eta squared effect size of 0.12 (f =  0.37) for the primary outcome, which was the height in the SLDJ between conditions and time. Consequently, a total of 22 participants were needed for the study, considering a possible 10% dropout rate.

### Participants

The inclusion criteria were (1) males and females (2) between 18 – 45 years old (3) well-trained, with a weekly training schedule of at least two days and a total of 20 km of running. The exclusion criteria were determined by the presence of musculoskeletal lower limb or lumbopelvic pathology in the last year, such as ankle sprains, patellofemoral pain syndrome, Achilles tendinopathy, or lumbar disc herniation. Participants were recruited through a call for volunteers via posters placed at the European University.

**Fig 1:**
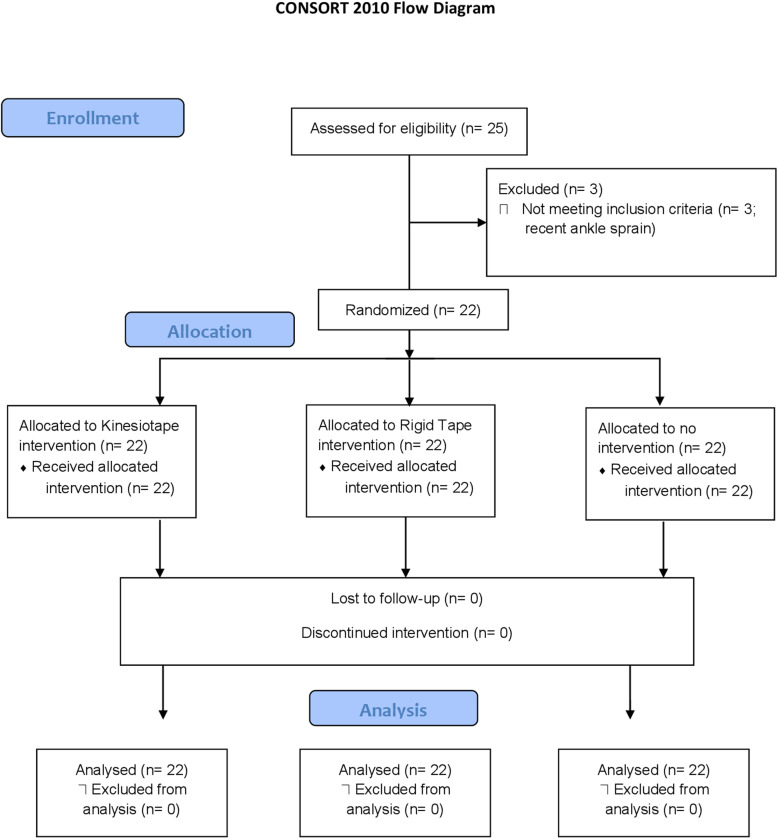
CONSORT Flow Diagram.

Initially, 25 individuals were assessed for eligibility, and 3 were excluded due to recent ankle sprains (Fig 1).

### Blinding and randomization

The investigator who performed the statistical analysis was blinded by receiving anonymized datasets, which were coded without identifiers indicating group allocation. This ensured that the analysis was conducted without bias regarding the participants’ group assignments. Participants were randomly assigned to a sequence of interventions (no tape, KT, and RT) using a computer-generated randomization sequence. This ensured that each participant underwent all three conditions in a random order, minimizing potential order effects and carryover bias. Because of the nature of the intervention, there was no blinding of the participants or the physiotherapist who performed the taping.

### Sociodemographic variables

Athlete’s gender (male or female), age (years), height (cm), weight (kg), Body Mass Index (BMI) (kg/cm2), dominant limb (right or left), and history of ankle sprains were collected as descriptive data. In addition, the following weekly training characteristics were collected: number of training days, running distance (km), mean pace (min/km), presence of specific lower limb and core muscle strength, or proprioceptive training (Table 1).

**Table 1:** Sociodemographic data of the sample.

Data	Total Sample (N = 22)	Female (n = 7)	Male (n = 15)
Age (years)	29.29 ± 10.98	31.20 ± 15.35	28.22 ± 8.63
Weight (kg)	66.14 ± 8.17	57.10 ± 3.21	72.20 ± 5.28
Height (m)	1.74 ± 0.06	1.69 ± 0.05	1.78 ± 0.04
BMI (kg/m^2^)	21.83 ± 1.67	20.10 ± 1.14	22.8 ± 1.34
Test distance 5min (m)	1172 ± 167	1064 ± 82	1256 ± 168
Aerobic Maximal Speed, (m/s)	3.90 ± 0.55	3.54 ± 0.27	4.18 ± 0.56
Velocity Fatigue Protocol (km/h)	11.90 ± 1.69	10.80 ± 0.73	12.8 ± 1.71
Training Week Volume (km)	27.30 ± 10.6	20.80 ± 7.52	31.7 ± 10.2
Lower Limb Length (cm)	103 ± 4.72	99.80 ± 4.26	105 ± 3.95
90º Squat Height (cm)	76.10 ± 4.40	75.30 ± 4.27	76.70 ± 4.48

### Intervention protocol

Voluntary participants visited the study center on Day 1 for the assessment of eligibility criteria, collection of demographic data, and completion of a maximal effort 5-minute run test on a 400-m track [32] to determine their individual maximal aerobic speed (MAS). The total distance covered was measured and MAS was calculated by dividing the total distance by the duration of the test. This allowed an accurate and individualised determination of the participants’ aerobic capacity, which was then used to establish the intensity of the treadmill fatigue protocol.

Subsequently, participants were scheduled for three sessions (once a week) to undergo the intervention under three different conditions, with the order randomized: no tape (Control Group), one with RT bandage [33], and another with KT [34]. The randomization of the participants was carried out with the random function of Microsoft Office Excel (Microsoft Corporation, Redmond, WA, USA). The bandaging was consistently applied to the dominant leg by a physiotherapist with ten years of clinical experience specializing in ankle and foot injuries in runners, within the Sports Performance Laboratory of the European University of Madrid.

A 10-minute warm-up was performed based on lower limb mobility exercises, ballistic stretching and running. Subsequently, an assessment was conducted by a different investigator than the one who applied the taping, and it included:

- ***Lower limb stability***: The Y balance test [35] was used to analyze the stability of the dominant lower limb. Subjects stood in the center of the Y-shaped area. While balancing on one leg, they strove to reach as far as possible with the other limb without falling along each of the three branches of the Y: anterior, posterior-right and posterior-left. Measurements were taken from the tip of the foot in each direction, with three trials conducted to obtain a mean [36].
- ***Active ankle dorsiflexion mobility*** was measured through the MyROM app during the Lunge test. In a knight’s stance, with the dominant limb on the ground and hands on the waist, participants were instructed to lean forward as far as possible without taking off the heel [37,38]. A mobile phone was placed along the tibia to track the dorsiflexion angle, and measurements were recorded on the screen. Each participant underwent three measurements and the mean between measurements was calculated [39]*.*
- ***Electromyographic activation of the ankle muscles*** was measured during the SLDJ, in the following phases: first contact, takeoff, and second contact. The minimal peak, mean, and maximal peak values were evaluated. To assess EMG activation, the tibialis anterior (TA), extensor digitorum longus (EDL), peroneus longus (PL), and medial gastrocnemius (MG) were evaluated using the EMG analysis equipment Delsys (Trigno Avanti; Natick, USA), assessing the total mean and ratios of contraction [40].

**Fig 2:**
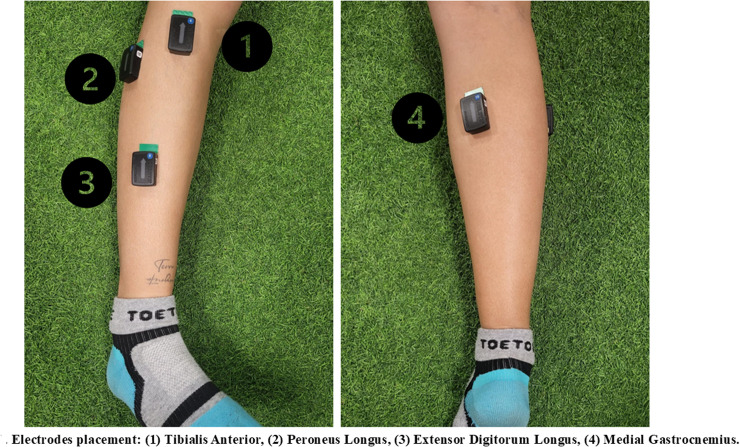
Electrodes placement: (1) Tibialis Anterior, (2) Peroneus Longus, (3) Extensor Digitorum Longus, (4) Medial Gastrocnemius.

For skin preparation and electrode application and placement, the guidelines outlined in the Surface Electromyography for the Non-Invasive Assessment of Muscles (SENIAM) were followed. (1) TA: The electrode is positioned on the front of the leg, over the muscle belly. The midpoint is approximately one-third of the distance between the tibia and the outer edge. (2) EDL: The electrode is also placed at the front of the leg, over the muscle belly. The midpoint of the muscle is one-third of the distance between the tibia and the outer edge. (3) PL: The electrode is situated in the lateral region of the leg, on the muscle belly. The midpoint is located at one-third of the distance between the tibia and the external border. (4) MG: The electrode is positioned on the back of the leg, over the muscle belly. The midpoint is approximately one-third of the distance between the bottom of the calf and the top of the heel (Fig 2).

To normalize the EMG signal, the activation of each muscle was expressed as a percentage of its maximum voluntary isometric contraction (MVIC). MVIC tests were performed for each muscle by asking participants to perform a standardized isometric contraction against resistance for 5 seconds, and 3 times. The highest EMG amplitude recorded over a 1-second interval during these trials was used as the reference value for normalization. This approach ensures comparability between participants and conditions. These evaluations were conducted on the first day of the running protocol (Day 2). Participants executed three isometric contractions against resistance for 5 seconds, with 15 seconds of rest between repetitions. For the TA, subjects assumed a seated position and performed dorsiflexion, adduction, and supination. For the EDL, participants, in a seated position, executed pure eversion. The PL was assessed with participants in a seated position, performing plantarflexion, abduction, and pronation. The MVIC assessment of the MG involved participants lying prone with the knee extended, performing inversion.

- ***Jumping ability*** was assessed using the SLDJ test, with the dominant leg being used for all trials to ensure consistency with other measures. Each participant performed three jumps on the dominant leg, and the best quality jump was selected for analysis. The characteristics of the jump were analyzed with the MyJump 2 app [41]. This test consists of a participant standing on one leg on a raised platform and then quickly dropping down and immediately jumping vertically with the same leg. The MyJump 2 app allows for the collection of several key variables, including:
  - Jump Height: Measuring the vertical displacement from the initial jump.
  - Flight Time: The duration the participant spends in the air during the jump.
  - Contact Time: The time the participant’s foot is in contact with the ground during the jump.
  - Reactive Strength Index (RSI): Calculated as the ratio of jump height to contact time, providing insights into the efficiency of the jump.
  - Stiffness (kN/m): The resistance of the lower limbs to deformation under load during activities such as jumping or running.

Following this evaluation, a fatigue protocol was implemented, involving a 30-minute run at 85% of the MAS on a treadmill [32]. The intensity of the run was individually tailored for each participant, based on their previously determined MAS, ensuring that each participant ran at 85% of their maximal aerobic capacity. This approach allowed for a standardized yet individualized intensity, ensuring consistency in the fatigue protocol while accounting for differences in fitness levels across participants [32].

**Fig 3:**
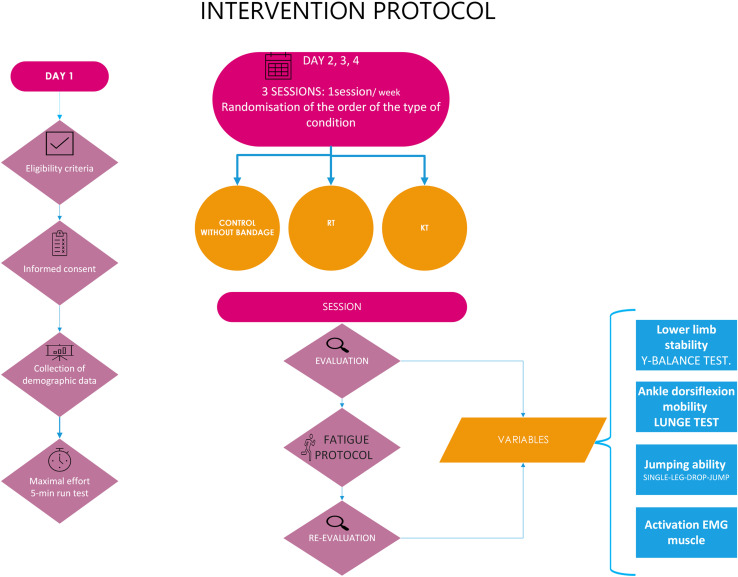
Flowchart of the study.

Once completed, an immediate reevaluation was conducted for all previously administered tests: Y-balance test, lunge test, and the SLDJ test, to collect the same variables as at the beginning of the running protocol (Fig 3).

### Procedure

#### KT Bandage.

**Fig 4:**
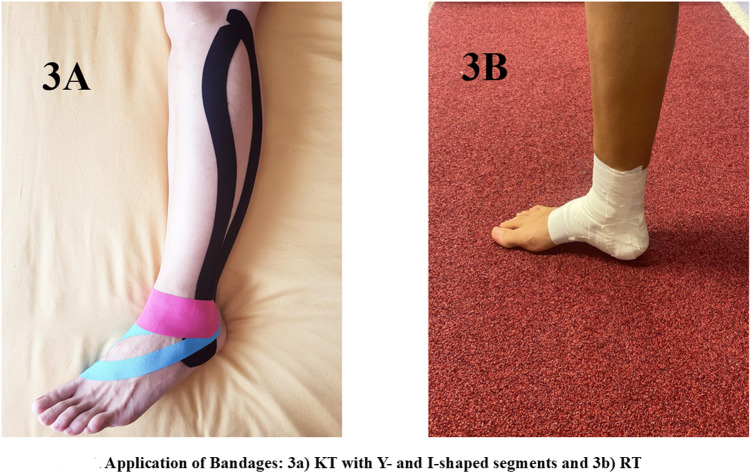
Bandages: KT (3A) and RT (3B).

In the KT bandage application (Fig 4), three coloured segments of elastic KT were employed: two Y-shaped segments and one I-shaped segment. First, the longer black Y-shaped segment was applied. First, the longer black Y-shaped segment was applied, beginning at the lateral aspect of the calcaneus and extending to the fibular head, covering the PL muscle. Next, a blue, shorter Y-shaped segment was applied from the posterior aspect of the calcaneus to the base of the first metatarsophalangeal joint. Finally, the pink I-shaped segment was applied longitudinally, extending from the tibial malleolus to the peroneal malleolus and crossing the anterior portion of the ankle. All segments were applied with a minimum tension of no more than 25%.

#### RT Bandage.

The ankle RT procedure was executed utilizing a conventional 38 mm self-adhesive tape, initiating with the application of two anchor straps at a position approximately 10 cm proximal to the malleoli. This was followed by the strategic placement of two additional straps extending from the medial edge of the anchor strap to the lateral side, with the foot maintained in a neutral position. Subsequently, “figure six” configurations were crafted, commencing with a strip originating from the medial anchor, passing through the plantar aspect of the foot, and reattaching to the medial anchor. To finalize the taping ankle procedure, the practitioner meticulously covered all terminal ends and any laxity with adhesive tape, ensuring a secure and uniform application (Fig 4).

### Statistical analysis

The statistical analysis was performed with the SPSS software version 29 for Windows. First, data distribution was assessed with the Shapiro-Wilk test. Then, a one-way ANOVA was performed to analyze the difference between conditions in pre-fatigue. A two-way repeated measures ANOVA (3 x 2; 3 conditions and 2 times of measurement) was employed to assess the effects of the intervention pre and post fatigue treadmill protocol, with the group factor treated as a fixed effect and time treated as a random effect to account for individual variability across repeated measures. Levene’s test was used to assess the homogeneity of variances between conditions, and the Greenhouse-Geisser correction was applied when the assumption of sphericity was violated. Effect sizes were estimated using partial eta squared (η²p), with values interpreted as small (0.01), medium (0.06), and large (0.14). All variables were analyzed individually, and multiple comparisons were accounted for using a Bonferroni correction. The percentage change was calculated between the pre-fatigue and post-fatigue measurements using the formula: Percentage Change =  [(Post-fatigue Value – Pre-fatigue Value)/ Pre-fatigue Value] ×  100, with the corresponding 95% confidence interval (CI). The significance level was set at 0.05.

## Results

Table 1 shows the anthropometric data (age, weight, height, BMI, lower limb length and squat height at 90º of hip flexion) and performance parameters of the total sample. A total of n =  22 well trained runners aged 29.29 ±  10.98 years, with a BMI of 21.83 ±  1.67 completed the study.

Table 2 shows the pre and post fatigue treadmill protocol measurements of the lunge test, SLDJ, flight and ground contact time, stiffness and, anterior, posteromedial and posterolateral measurements of the Y-balance test. Table 3 shows the pre-fatigue and post-fatigue measurements of the EMG activation of the PL, TA, ELD, and GM muscles.

**Table 2:** ANOVA repeated measures of the variables.

	3 CONDITIONS N = 22					
	**CONTROL GROUP**	**KT**	**RT**	**Time value** **F; P (Eta2)**	**Group value** **F; P (Eta2)**	**Group X Time** **F; P (Eta2)**
Lunge (degrees)						
Pre-fatigue	45.41 ± 5.68	43.80 ± 5.13	39.71 ± 4.24	F = 3.04; P = 0.086 (0.048)	F = 4.51; P = 0.015* (0.131)	F = 3.27; P = 0.045* (0.098)
Post-fatigue	45.45 ± 5.96	43.70 ± 5.40	42.05 ± 5.14			
Percentage change; mean difference (95%CI)	0.09%; 0.04 (95% CI: ‒0.86, 0.94)	‒0.23% change; ‒0.10 (95% CI: ‒0.99, 0.79)	5.89% change; 2.34 (95% CI: 1.38, 3.30)			
Reactive Force Index						
Pre-fatigue	0.94 ± 0.34	0.96 ± 0.25	0.93 ± 0.25	F = 0.25; P = 0.617 (0.004)	F = 0.044; P = 0.957 (0.001)	F = 0.870; P = 0.424 (0.028)
Post-fatigue	0.93 ± 0.28	0.96 ± 0.28	0.98 ± 0.27			
Percentage change; mean difference (95%CI)	‒1.06% change; ‒0.01 (95% CI: ‒0.12, 0.10)	0.00% change; 0.00 (95% CI: ‒0.09, 0.09)	5.38% change; 0.05 (95% CI: ‒0.04, 0.14)			
SLDJ (cm)						
Pre-fatigue	17.04 ± 6.55	16.29 ± 5.91	14.34 ± 5.66	F = 0.06; P = 0.805 (0.001)	F = 0.277; P = 0.759 (0.009)	F = 4.566; P = 0.029* (0.108)
Post-fatigue	15.13 ± 5.79	16.40 ± 6.78	15.79 ± 6.02			
Percentage change; mean difference (95%CI)	‒11.20% change; ‒1.91 (95% CI: ‒3.64, ‒0.18)	0.68% change; 0.11 (95% CI: ‒1.61, 1.83)	10.10% change; 1.45 (95% CI: ‒0.18, 3.08)			
Flight time (ms)						
Pre-fatigue	365.73 ± 73.65	357.85 ± 66.17	335.62 ± 66.21	F = 0.03; P = 0867 (0.000)	F = 0.248; P = 0.781 (0.008)	F = 4.236; P = 0.019* (0.124)
Post-fatigue	344.95 ± 68.14	358.20 ± 75.57	353.29 ± 65.38			
Percentage change; mean difference (95%CI)	‒5.67% change; ‒20.78 (95% CI: ‒42.27, 0.71)	0.10% change; 0.35 (95% CI: ‒21.25, 21.95)	5.26% change; 17.67 (95% CI: ‒3.67, 39.01)			
Ground Contact Time (ms)						
Pre-fatigue	416.55 ± 105.67	381.05 ± 74.07	370.86 ± 63.06	F = 1.59; P = 0.212 (0.026)	F = 0.346; P = 0.709 (0.011)	F = 3.556; P = 0.035* (0.106)
Post-fatigue	365.76 ± 106.44	390.70 ± 95.46	373.33 ± 64.37			
Percentage change; mean difference (95%CI)	‒12.18% change; ‒50.79 (95% CI: ‒87.74, ‒13.84)	2.53% change; 9.65 (95% CI: ‒27.30, 46.60)	0.67% change; 2.47 (95% CI: ‒34.37, 39.31)			
Stiffness (kN/m)						
Pre-fatigue	7.40 ± 2.87	8.17 ± 2.88	8.62 ± 2.45	F = 0.87; P = 0.355 (0.014)	F = 0.509; P = 0.604 (0.017)	F = 1.071; P = 0.349 (0.034)
Post-fatigue	8.10 ± 2.53	8.17 ± 3.46	8.58 ± 3.21			
Percentage change; mean difference (95%CI)	9.46% change; 0.70 (95% CI: ‒0.43, 1.83)	0.00% change; 0.00 (95% CI: ‒1.04, 1.04)	‒0.46% change; ‒0.04 (95% CI: ‒0.93, 0.85)			
YBT-A (cm)						
Pre-fatigue	58.70 ± 7.03	58.88 ± 5.95	53.93 ± 6.90	F = 0.014; P = 0.905 (0.000)	F = 3.638; P = 0.032* (0.108)	F = 0.142; P = 0.868 (0.005)
Post-fatigue	58.34 ± 7.60	59.23 ± 7.83	53.74 ± 7.90			
Percentage change; mean difference (95%CI)	‒0.61% change; ‒0.36 (95% CI: ‒2.18, 1.46)	0.59% change; 0.35 (95% CI: ‒1.39, 2.09)	‒0.35% change; ‒0.19 (95% CI: -2.12, 1.74)			
YBT-PM (cm)						
Pre-fatigue	94.18 ± 10.25	93.35 ± 6.15	93.29 ± 7.11	F = 0.11 P = 0.735 (0.002)	F = 0.145; P = 0.865 (0.005)	F = 0.448; P = 0.641 (0.015)
Post-fatigue	91.41 ± 17.96	94.70 ± 7.59	92.88 ± 8.03			
Percentage change; mean difference (95%CI)	‒2.94% change; ‒2.77 (95% CI: ‒8.37, 2.83)	1.45% change; 1.35 (95% CI: ‒1.77, 4.47)	‒0.44% change; ‒0.41 (95% CI: ‒3.07, 2.25)			
YBT-PL (cm)						
Pre-fatigue	98.36 ± 9.81	97.65 ± 6.29	94.95 ± 9.52	F = 3.86; P = 0.054 (0.060)	F = 0.586; P = 0.560 (0.019)	F = 0.404; P = 0.669 (0.013)
Post-fatigue	95.86 ± 10.26	96.45 ± 8.81	94.05 ± 11.07			
Percentage change; mean difference (95%CI)	‒2.54% change; ‒2.50 (95% CI: ‒6.57, 1.57)	‒1.23% change; ‒1.20 (95% CI: ‒4.24, 1.84)	‒0.95% change; ‒0.90 (95% CI: ‒4.00, 2.20)			

Abbreviatures: CI, Confidence Interval; SLDJ, Single Leg Drop Jump; KT, Kinesiotape; RT, Rigid Tape * Significance was set at p < 0.05.

**Table 3:** ANOVA repeated measures of the electromyographic muscle activation.

MUSCLE	MOMENT	VARIABLE	CONDITIONS	PRE-FATIGUE	POST - FATIGUE	Time value F; P (η ^2^)	Group value F; P (η ^2^)	Group X Time F; P (η ^2^)
PERONEUS LONGUS	FIRST CONTACT	PEAK MIN	CONTROL GROUP	3.65 ± 2.55	6.10 ± 6.23	F = 0.109; P = 0.742 (0.002)	F = 3.547; P = 0.035* **(0.104)**	F = 1.182; P = 0.314 (0.037)
			KT	14.71 ± 23.96	8.38 ± 21.55			
			RT	4.43 ± 3.76	5.81 ± 6.70			
		MEAN	CONTROL GROUP	27.59 ± 20.81	24.44 ± 17.39	F = 0.206; P = 0.651 (0.003)	F = 3.231; P = 0.046* **(0.096)**	F = 0.009; P = 0.991 (0.000)
			KT	47.06 ± 46.66	42.40 ± 80.86			
			RT	25.92 ± 16.92	23.60 ± 24.81			
		PEAK MAX	CONTROL GROUP	76.23 ± 95.05	63.08 ± 49.80	F = 0.651; P = 0.423 (0.011)	F = 2.725; P = 0.074 (0.082)	F = 0.002; P = 0.998 (0.000)
			KT	119.21 ± 136.66	103.76 ± 171.22			
			RT	68.91 ± 63.97	53.38 ± 55.27			
	TAKE OFF	PEAK MIN	CONTROL GROUP	22.87 ± 13.80	21.50 ± 19.44	F = 0.125; P = 0.725 (0.002)	F = 0.446; P = 0.642 (0.014)	F = 0.269; P = 0.765 (0.009)
			KT	25.42 ± 22.24	27.32 ± 29.03			
			RT	24.30 ± 22.37	20.29 ± 16.30			
		MEAN	CONTROL GROUP	59.93 ± 29.50	49.36 ± 29.43	F = 1.420; P = 0.238 (0.023)	F = 2.909; P = 0.062 (0.087)	F = 0.191; P = 0.827 (0.006)
			KT	119.21 ± 136.66 79.50 ± 45.78	75.72 ± 47.06 60.89 ± 33.02			
			RT					
		PEAK MAX	CONTROL GROUP	101.25 ± 49.86	94.93 ± 54.53	F = 0.604; P = 0.440 (0.010)	F = 2.583; P = 0.084 (0.078)	F = 0.926; P = 0.402 (0.029)
			KT	154.77 ± 101.29	124.36 ± 66.83			
			RT	112.27 ± 50.24	121.20 ± 108.87			
	SECOND CONTACT	PEAK MIN	CONTROL GROUP	13.44 ± 11.62	15.05 ± 22.18	F = 0.340; P = 0.562 (0.006)	F = 0.438; P = 0.647 (0.014)	F = 0.028; P = 0.972 (0.001)
			KT	14.00 ± 12.10	15.83 ± 25.48			
			RT	10.83 ± 10.57	11.41 ± 14.34			
		MEAN	CONTROL GROUP	40.72 ± 37.03	58.36 ± 76.48	F = 0.028; P = 0.868 (0.000)	F = 1.055; P = 0.354 (0.033)	F = 0.164; P = 0.849 (0.005)
			KT	73.28 ± 97.12	68.18 ± 67.22			
			RT	48.09 ± 30.38	49.42 ± 56.03			
		PEAK MAX	CONTROL GROUP	92.90 ± 77.88	152.36 ± 272.67	F = 0.832; P = 0.365 (0.013)	F = 0.936; P = 0.398 (0.030)	F = 0.442; P = 0.645 (0.014)
			KT	172.89 ± 257.58	166.80 ± 166.90			
			RT	98.30 ± 69.62	122.26 ± 183.37			
MEDIAL GASTROCNEMIUS	FIRST CONTACT	PEAK MIN	CONTROL GROUP	9.82 ± 12.75	7.86 ± 13.18	F = 0.703; P = 0.405 (0.011)	F = 0.170; P = 0.844 (0.006)	F = 0.294; P = 0.746 (0.010)
			KT	11.68 ± 11.67	9.23 ± 12.87			
			RT	9.13 ± 9.01	9.49 ± 6.98			
		MEAN	CONTROL GROUP	48.60 ± 55.00	43.47 ± 42.63	F = 1.220; P = 0.274 (0.020)	F = 0.716; P = 0.493 (0.023)	F = 0.499; P = 0.609 (0.016)
			KT	75.23 ± 126.43	47.72 ± 64.87			
			RT	46.81 ± 31.78	43.27 ± 28.69			
		PEAK MAX	CONTROL GROUP	100.45 ± 110.05	93.00 ± 105.69	F = 1.221; P = 0.273 (0.020)	F = 0.582; P = 0.562 (0.019)	F = 0.767; P = 0.469 (0.025)
			KT	164.87 ± 300.21	92.97 ± 113.43			
			RT	97.64 ± 82.57	93.64 ± 84.85			
	TAKE OFF	PEAK MIN	CONTROL GROUP	28.92 ± 34.60	17.45 ± 17.69	F = 2.681; P = 0.107 (0.042)	F = 0.590; P = 0.558 (0.019)	F = 0.706; P = 0.498 (0.023)
			KT	48.54 ± 121.58	18.44 ± 24.23			
			RT	23.32 ± 20.67	19.40 ± 20.55			
		MEAN	CONTROL GROUP	87.27 ± 52.43	60.54 ± 38.51	F = 2.253; P = 0.138 (0.036)	F = 0.751; P = 0.476 (0.024)	F = 0.176; P = 0.839 (0.006)
			KT	110.76 ± 170.04	83.01 ± 87.71			
			RT	83.16 ± 45.37	73.63 ± 44.65			
		PEAK MAX	CONTROL GROUP	164.29 ± 103.67	113.21 ± 79.44	F = 1.290; P = 0.261 (0.021)	F = 0.288; P = 0.751 (0.009)	F = 0.591; P = 0.557 (0.019)
			KT	173.94 ± 212.07	149.47 ± 170.16			
			RT	142.38 ± 78.40	146.73 ± 91.37			
	SECOND CONTACT	PEAK MIN	CONTROL GROUP	7.30 ± 6.82	7.01 ± 5.09	F = 0.190; P = 0.665 (0.003)	F = 0.412; P = 0.664 (0.013)	F = 2.016; P = 0.142 (0.062)
			KT	8.21 ± 9.61	11.09 ± 17.84			
			RT	10.66 ± 13.47	6.06 ± 6.29			
		MEAN	CONTROL GROUP	43.79 ± 32.64	41.60 ± 49.86	F = 0.583; P = 0.448 (0.009)	F = 1.512; P = 0.229 (0.047)	F = 0.081; P = 0.922 (0.003)
			KT	65.43 ± 86.07	57.89 ± 73.13			
			RT	48.65 ± 33.59	37.61 ± 22.39			
		PEAK MAX	CONTROL GROUP	94.72 ± 60.76	90.98 ± 111.30	F = 0.004; P = 0.951 (0.000)	F = 3.097; P = 0.052 (0.092)	F = 0.001; P = 0.999 (0.000)
			KT	176.15 ± 239.34	175.19 ± 285.39			
			RT	109.34 ± 84.91	108.43 ± 79.31			
EXTENSOR DIGITUS	FIRST CONTACT	PEAK MIN	CONTROL GROUP	3.04 ± 2.61	4.49 ± 4.41	F = 0.720; P = 0.399 (0.012)	F = 1.247; P = 0.295 (0.039)	F = 1.180; P = 0.314 (0.037)
			KT	13.83 ± 41.57	4.62 ± 5.03			
			RT	4.56 ± 3.69	4.53 ± 3.92			
		MEAN	CONTROL GROUP	27.21 ± 21.33	22.12 ± 13.78	F = 0.796; P = 0.376 (0.013)	F = 0.876; P = 0.422 (0.028)	F = 2.091; P = 0.132 (0.064)
			KT	47.54 ± 57.54 24.97 ± 19.79	39.22 ± 62.65 71.60 ± 168.36			
			RT					
		PEAK MAX	CONTROL GROUP	73.74 ± 62.03	60.11 ± 49.53	F = 1.207; P = 0.276 (0.019)	F = 1.172; P = 0.316 (0.037)	F = 3.793; P = 0.028* **(0.111)**
			KT	128.23 ± 169.55	96.93 ± 137.86			
			RT	61.97 ± 58.92	196.69 ± 352.42			
	TAKE OFF	PEAK MIN	CONTROL GROUP	11.21 ± 11.62	11.06 ± 7.48	F = 0.227; P = 0.636 (0.004)	F = 0.944; P = 0.395 (0.030)	F = 4.417; P = 0.016* **(0.127)**
			KT	17.39 ± 18.59	10.52 ± 9.04			
			RT	13.62 ± 12.14	18.35 ± 16.90			
		MEAN	CONTROL GROUP	29.13 ± 22.85	28.76 ± 18.90	F = 2.551; P = 0.115 (0.040)	F = 2.713; P = 0.074 (0.082)	F = 2.567; P = 0.085 (0.078)
			KT	47.88 ± 45.57	48.39 ± 62.79			
			RT	37.94 ± 22.22	81.93 ± 109.71			
		PEAK MAX	CONTROL GROUP	58.70 ± 43.13	61.36 ± 39.65	F = 2.838; P = 0.097 (0.044)	F = 2.931; P = 0.061 (0.088)	F = 1.699; P = 0.191 (0.053)
			KT	112.47 ± 96.33	132.45 ± 268.19			
			RT	92.24 ± 90.81	211.46 ± 278.32			
	SECOND CONTACT	PEAK MIN	CONTROL GROUP	10.25 ± 10.68	9.89 ± 10.15	F = 0.246; P = 0.622 (0.004)	F = 0.806; P = 0.451 (0.026)	F = 0.597; P = 0.553 (0.019)
			KT	9.29 ± 7.29	8.83 ± 12.75			
			RT	11.28 ± 12.11	14.35 ± 15.54			
		MEAN	CONTROL GROUP	47.88 ± 39.52	50.69 ± 68.25	F = 0.741; P = 0.393 (0.012)	F = 1.878; P = 0.162 (0.058)	F = 1.773; P = 0.178 (0.055)
			KT	102.77 ± 144.18	94.74 ± 109.70			
			RT	54.09 ± 53.70	116.18 ± 200.95			
		PEAK MAX	CONTROL GROUP	105.57 ± 98.59	116.95 ± 124.45	F = 1.057; P = 0.308 (0.017)	F = 2.542; P = 0.087 (0.077)	F = 1.170; P = 0.317 (0.037)
			KT	307.56 ± 480.58	291.92 ± 403.94			
			RT	144.92 ± 172.27	327.68 ± 569.20			
ANTERIOR TIBIALIS	FIRST CONTACT	PEAK MIN	CONTROL GROUP	4.35 ± 5.03	4.59 ± 5.26	F = 0.160; P = 0.690 (0.003)	F = 0.778; P = 0.464 (0.025)	F = 0.802; P = 0.453 (0.032)
			KT	7.85 ± 11.18	5.23 ± 8.98			
			RT	4.84 ± 4.01	4.76 ± 6.66			
		MEAN	CONTROL GROUP	49.34 ± 77.81	36.51 ± 40.69	F = 0.016; P = 0.901 (0.000)	F = 1.544; P = 0.222 (0.048)	F = 0.194; P = 0.824 (0.006)
			KT	59.67 ± 81.57	76.03 ± 24.58			
			RT	24.06 ± 15.92	27.65 ± 22.84			
		PEAK MAX	CONTROL GROUP	162.67 ± 301.77	115.99 ± 143.11	F = 0.000; P = 0.986 (0.000)	F = 1.502; P = 0.231 (0.047)	F = 0.226; P = 0.798 (0.007)
			KT	177.59 ± 250.12 53.87 ± 31.12	191.20 ± 589.18 84.37 ± 95.67			
			RT					
	TAKE OFF	PEAK MIN	CONTROL GROUP	31.33 ± 102.75	10.74 ± 8.76	F = 0.975; P = 0.327 (0.016)	F = 0.367; P = 0.695 (0.012)	F = 0.661; P = 0.520 (0.021)
			KT	18.97 ± 26.08	17.35 ± 28.73			
			RT	13.30 ± 10.47	12.22 ± 10.48			
		MEAN	CONTROL GROUP	72.32 ± 175.50	32.35 ± 29.68	F = 1.396; P = 0.242 (0.022)	F = 0.505; P = 0.606 (0.016)	F = 0.546; P = 0.582 (0.018)
			KT	74.59 ± 107.19	55.39 ± 110.86			
			RT	41.72 ± 43.39	43.22 ± 36.35			
		PEAK MAX	CONTROL GROUP	169.64 ± 402.90	87.88 ± 102.43	F = 1.407; P = 0.240 (0.023)	F = 0.340; P = 0.713 (0.011)	F = 0.685; P = 0.508 (0.022)
			KT	180.93 ± 236.31	116.18 ± 257.27			
			RT	96.28 ± 110.46	112.22 ± 114.62			
	SECOND CONTACT	PEAK MIN	CONTROL GROUP	11.15 ± 18.29	10.45 ± 10.25	F = 0.137; P = 0.712 (0.002)	F = 0.209; P = 0.812 (0.007)	F = 0.220; P = 0.803 (0.007)
			KT	13.04 ± 21.97	13.23 ± 22.77			
			RT	9.28 ± 7.97	13.07 ± 16.99			
		MEAN	CONTROL GROUP	91.61 ± 145.60	59.32 ± 65.92	F = 2.476; P = 0.121 (0.039)	F = 1.157; P = 0.321 (0.037)	F = 0.895; P = 0.414 (0.029)
			KT	113.56 ± 119.81 56.45 ± 79.12	74.90 ± 88.31 60.31 ± 38.06			
			RT					
		PEAK MAX	CONTROL GROUP	235.43 ± 291.46	164.99 ± 213.19	F = 2.560; P = 0.115 (0.040)	F = 0.914; P = 0.406 (0.029)	F = 0.334; P = 0.717 (0.011)
			KT	291.04 ± 325.42	186.07 ± 248.01			
			RT	164.74 ± 339.55	141.79 ± 96.97			

*Significance was set at p < 0.05.

Pre-fatigue measurements revealed significant differences between the RT group and the control group (p =  0.001, mean difference =  5.70º, 95% CI [3.12º, 8.28º]), as well as the KT Group (p =  0.037, mean difference =  3.09º, 95% CI [0.17º, 6.01º]) for the lunge test. For all other variables, there were no significant differences in pre-fatigue (p >  0.05). Group interaction analysis showed significant differences for the lunge test (p =  0.015, mean difference =  2.72º, 95% CI [0.80º, 4.64º]) and anterior Y-balance test (p =  0.032, mean difference =  4.41 cm, 95% CI [0.38 cm, 8.44 cm]). Pairwise comparison showed significant differences (mean difference =  5.70º; p =  0.001, 95% CI [3.12º, 8.28º]) between the control group and RT group in pre-fatigue.

No significant differences were observed in the time effect for all the variables. Group-by-time interaction showed significant differences for the lunge test (p =  0.045, mean difference =  2.33º, 95% CI [0.09º, 4.57º]), SLDJ height (p =  0.014, mean difference =  1.90 cm, 95% CI [0.36 cm, 3.44 cm]), flight time (p =  0.019, mean difference =  20.77ms, 95% CI [2.34ms, 39.20ms]) and ground contact time (p =  0.035, mean difference =  50.79ms, 95% CI [16.39ms, 85.19ms]). No significant differences were observed for the rest of the variables (p >  0.05). Pairwise comparison showed significant differences between the pre-fatigue and post-fatigue measurements in the RT group for the lunge test (mean difference =  2.33º; p =  0.003, 95% CI [0.81º, 3.85º]), and in the control group for the SLDJ height (mean difference =  1.90 cm; p =  0.018, 95% CI [0.29 cm, 3.51 cm]); flight time (mean difference =  20.77ms; p =  0.028, 95% CI [2.34ms, 39.20ms]), and ground contact time (mean difference =  50.79ms; p =  0.005, 95% CI [16.39ms, 85.19ms]) (Table 2). No significant differences were observed in the KT group (p >  0.05).

Concerning the EMG analysis, group interaction analysis showed significant differences between the KT group and the other groups for the PL activation during the first contact (peak min, p =  0.035; mean, p =  0.046). In addition, the group by time interaction showed significant differences between the RT group and the other groups for the EDL during the first contact (peak max, p =  0.028) and the takeoff phase (peak min, p =  0.016). There were no significant differences in the time effect for all the variables (p >  0.05) (Table 3).

For the first contact phase, in the peak min activation, the cg showed a substantial increase of 67.12%, with a mean difference of 2.45 (95% ci: -0.54, 5.44). The KT group, in contrast, demonstrated a decrease of 43.05%, with a mean reduction of -3.15 (95% ci: -12.98, 6.68), while the rt group experienced a moderate increase of 31.11%, with a mean difference of 1.38 (95% ci: -2.42, 5.18). For the mean activation, the cg saw a reduction of 11.41%, with a mean change of -3.15 (95% ci: -12.98, 6.68). The KT group exhibited a similar decrease of 9.92% with a mean difference of -4.67 (95% ci: -18.10, 8.76), and the rt group reduced by 8.96%, with a mean difference of -2.32 (95% ci: -11.56, 6.92). In peak max activation, the cg showed a decrease of 17.28%, with a mean reduction of -13.15 (95% ci: -44.46, 18.16), while the KT and groups had declines of 13.19% and 22.55%, respectively, without significant changes noted. The KT group showed a mean difference of -15.45 (95% ci: -65.44, 34.54), and the rt group a difference of -15.53 (95% ci: -57.43, 26.37).

In the take-off phase, for peak min activation, the cg showed a small decrease of 5.98%, with a mean difference of -1.37 (95% ci: -8.18, 5.44), while the KT group decreased by 27.46% with a mean difference of -6.98 (95% ci: -19.56, 5.60), and the RT group showed a reduction of 16.51% with a mean difference of -4.01 (95% ci: -13.02, 5.00). For the mean activation, there was a reduction in cg at 17.47% (mean difference: -10.57; 95% ci: -24.32, 3.18), KT at 31.12% (mean difference: -24.49; 95% ci: -71.32, 22.34), and RT at 25.39% (mean difference: -20.78; 95% ci: -55.64, 14.08). Regarding peak max activation, the cg demonstrated a reduction of 6.19%, with a mean difference of -6.32 (95% ci: -40.58, 27.94), KT decreased by 16.08% (mean difference: -24.77; 95% ci: -60.00, 10.46), while the RT group showed a slight increase of 5.11% (mean difference: 7.19; 95% ci: -24.18, 38.56).

During the second contact phase, peak min activation showed minor changes, with the cg decreasing by 3.32% (mean difference: -0.28; 95% ci: -4.21, 3.65), KT increasing by 6.95% (mean difference: 0.64; 95% ci: -5.15, 6.43), and RT decreasing significantly by 43.16% (mean difference: -4.77; 95% ci: -13.25, 3.71). In the mean activation, the cg decreased by 4.58% with a mean difference of -2.04 (95% ci: -12.31, 8.23), KT by 11.63% with a mean difference of -7.54 (95% ci: -24.09, 9.01), and RT by 22.71% with a mean difference of -10.94 (95% ci: -30.25, 8.37). Finally, in the peak max activation, the cg showed a small reduction of 3.95%, with a mean difference of -3.74 (95% ci: -29.47, 21.99), KT decreased by 0.55%, with a mean difference of -0.96 (95% ci: -39.99, 38.07), and RT reduced by 1.53%, with a mean difference of -1.68 (95% ci: -53.56, 50.20).

In summary, the RT group showed significant differences in lunge test, CMJ test scores, Y-balance test scores, and muscle activation in the EDL. In contrast, the KT group exhibited significant muscle activation changes specifically in the PL muscles. These findings highlight the distinct effects of RT and KT on lower limb stability and muscle activation following fatigue.

## Discussion

This study aimed to test the hypothesis that the application of KT and RT bandage would have differential effects on lower limb stability, EMG activation of the pronator and supinator muscles of the ankle during a SLDJ, and ankle dorsiflexion ROM following a treadmill running fatigue protocol. The treadmill running protocol used in this study was designed to simulate real-world fatigue experienced during prolonged running. This protocol involved continuous treadmill running at 85% of the participant’s maximum oxygen uptake (VO2max) until exhaustion, replicating the fatigue conditions often encountered in competitive running events. Specifically, we hypothesized that the application of KT and RT bandage would have differential effects on lower limb stability, EMG activation of the pronator and supinator muscles of the ankle during a SLDJ, and active ankle dorsiflexion ROM, particularly following a treadmill running fatigue protocol.

### Ankle dorsiflexion ROM and YBT

The RT bandage reduced the ankle dorsiflexion ROM compared to KT bandage or Control group in pre-fatigue measurements during the lunge test and YBT (reduced anterior direction). Post-fatigue, this restriction in ankle dorsiflexion ROM was normalized (improved + 5.9%) relative to KT or Control group. Recently, Romero-Morales et al. [33] showed that a prophylactic ankle RT has a negative immediate effect on ankle dorsiflexion ROM, recommending against its use due to an associated increase in knee valgus during drop-jump tasks. Consistent with these findings, several studies have shown that limited ankle dorsiflexion ROM can negatively affect lower extremity mechanics during landing or sport-specific tasks, such as running, sprinting, or jumping [22,42–43]. Consequently, some authors advise against the use of tape bandages to prevent running-related fatigue in healthy individuals. However, Tang et al. [20] found that RT bandaging may provide benefits for individuals with chronic ankle instability, suggesting that taping should be considered selectively, depending on prior injuries or physical impairments.

These findings highlight a critical balance between restricting ankle motion to provide stability and ensuring adequate mobility to maintain optimal biomechanics. While RT may be beneficial for individuals with ankle instability, its restrictive nature could pose risks for healthy athletes by altering neuromuscular control and potentially increasing stress on other joints, such as the knee. Future studies should investigate whether long-term use of RT leads to compensatory movement patterns that could predispose individuals to secondary injuries.

Concerning the effects of KT application on YBT and ankle dorsiflexion ROM, no significant group-by-time interactions were observed between pre- and post-treadmill protocol. Unlike our findings, previous studies have shown that KT can improve ankle dorsiflexion ROM in duathletes [44] and mitigate fatigue effects on dynamic balance during the Modified Star Excursion Balance Test (SEBT) [45], particularly in the lateral and posterior directions. These discrepancies may stem from methodological differences (SEBT versus YBT) or differences in sample characteristics (duathletes versus recreational runners). Consistent with our findings, a cross-over clinical trial with 55 endurance athletes reported that KT did not prevent decreases in ankle dorsiflexion ROM after treadmill running [46]. Similarly, Kodesh and Dar assessed KT’s effect on dynamic stability following ankle muscles fatigue in people with chronic ankle instability, finding no significant effects during post-fatigue protocol [47]. Although KT has not demonstrated significant efficacy in reducing muscle fatigue following a running fatigue protocol, some studies suggest that it may have had positive outcomes in populations with ankle pathologies [48,49]. Thus, a previous systematic review concluded that current evidence does not support the routine use of KT [50].

Considering the mixed evidence regarding KT, it is possible that its effects are highly context-dependent. While some studies have reported benefits in specific athletic populations, our findings suggest that KT does not significantly influence ankle dorsiflexion ROM or dynamic balance in recreational runners. This underscores the importance of individualized taping strategies, where the specific needs of the athlete, their training background, and injury history should be considered. Future research should focus on identifying subgroups that may derive greater benefits from KT application.

### Single Leg Drop Jump

Regarding the SLDJ, participants of the KT group showed no significant differences in jump height when comparing pre- and post- fatigue protocol measurements. This suggests that KT may not mitigate ankle muscle fatigue in tasks such as the drop jump. In contrast, a recent meta-analysis provided evidence suggesting that KT can have positive effects on lower limb strength during vertical jumps [51]. However, research on the effects of KT application has yielded mixed results, with 54% of studies concluding that KT was ineffective, and 46% supporting its effectiveness [52,53].

In terms of flight and contact time, and height during the SLDJ, the RT bandage was beneficial for mitigating fatigue symptoms before and after the fatigue protocol ( + 10.1% increase in jump height). The control group exhibited shorter flight and contact times before the fatigue protocol compared to afterwards, whereas the RT group showed increased flight time post-fatigue relative to pre-fatigue. This increase could be due to the restrictive effects of tape bandage, which limits ankle dorsiflexion ROM and affects the anterior reach in the YBT. During landing from a drop-jump or single-leg counter movement jump (SL-CMJ), the joints and lower limbs must be primed for effective energy dissipation. Previous research has suggested that ankle joint restriction may reduce the lower limbs’ capacity to absorb ground reaction forces, potentially impairing performance in tasks like the drop-jump or SL-CMJ [54,55].

The observed effects of RT on jump performance suggest that while it may help mitigate certain fatigue symptoms, it does not necessarily enhance lower limb function in a way that translates to improved athletic performance. Instead, RT appears to provide mechanical support, potentially altering natural movement patterns. This raises important considerations regarding the trade-off between stability and dynamic performance. Athletes using RT should be aware of potential biomechanical compensations that could emerge over time, and future studies should explore how prolonged RT application influences neuromuscular adaptations.

The ability to jump or land is associated with enhanced athletics and reduced injury risk, particularly for athletes engaged in high-demand activities such as running. Thus, RT may help mitigate certain fatigue symptoms, such as reduced jump height, though it may not address stiffness, in athletes undergoing training that combines running with plyometric exercises. In practical terms, these findings suggest that RT should be used selectively based on the specific demands of the sport and the athlete’s injury profile. While it may serve as a useful tool for injury prevention in those with ankle instability, its application in healthy athletes should be approached with caution, as it may influence movement mechanics in unintended ways.

### Electromyography muscles activity

In this study, we hypothesized that the EMG activation of the ankle’s pronator and supinator muscles during the SLDJ would be significantly influenced by the different taping techniques. However, no significant differences were observed in TA or GM muscle activation across the conditions, time points, or specific phases of the SLDJ. This lack of significant differences may suggest that neither RT nor KT taping had a notable effect on the specific muscle activation required for the SLDJ. It is possible that the type of taping—whether KT or RT—did not substantially influence ankle muscle activation during this functional activity. Additionally, all participants performed the test wearing their usual footwear, which could have exerted a greater stabilizing effect than the bandage itself, potentially compensating for any changes induced by the taping. Supporting this interpretation, Slevin et al. [56] found that the transition from barefoot to shod conditions increased muscle activation in TA and PL, but observed no differences in response to the type of bandage.

With respect to the specific effects of KT on muscle activation, no significant changes were detected in TA, EDL, or GM activation when compared to Control group and RT groups. However, the PL muscle showed greater muscle activation during the first contact phase of the SLDJ (upon landing from the step) compared to the other conditions, independent of fatigue induced by the training protocol. The increase in PL activity during the first phase of the jump landing may reflect a protective response, serving as a preventative mechanism for ankle instability. Several studies have linked reduced PL muscle activation to chronic ankle instability [57–60]. For instance, Palmieri-Smith ([58] suggests that peroneal activity is decreased in functionally unstable ankles, which may contribute to recurrent joint instability and leave the ankle vulnerable to injurious loading. Consequently, the increased PL activation observed in the KT group suggests that this taping technique may enhance ankle stability during landing movements.

These findings suggest that KT may enhance neuromuscular control through proprioceptive stimulation, leading to increased preparatory muscle activation during critical phases of landing. This mechanism may be beneficial in reducing injury risk by facilitating pre-activation strategies that contribute to ankle stabilization. However, this hypothesis requires further investigation through studies incorporating proprioceptive assessments and movement variability analyses.

In line with the results found in the present research, several studies support the idea that these taping techniques can influence how the neuromuscular system responds to unexpected changes or alterations, potentially improving the stability and control of the ankle joint during physical activities [61]. It has been suggested that KT stimulates the proprioceptive reflex system connected to muscles via efferent nerves, which may lead to increased muscle activation. Some studies propose that KT improves joint position sense [62] by stimulating cutaneous mechanoreceptors. However, these results contradict the findings of Briem et al. [63], who found that the KT bandage did not affect the EMG activity of the PL muscle. This discrepancy can be attributed to the movement measured. In their study, they measured muscle activation during perturbations towards inversion, generated by the controlled fall of weight on the balance board, while in the present study, the investigators observed a jumping exercise with a very relevant landing component, where the PL muscle activation is essential for shock absorption and stabilization of the ankle. More studies evaluating different functional gestures are needed to delve deeper into the effects of KT and clarify these discrepancies.

Concerning the specific effects of RT bandage on muscle activation, in the present study the RT group showed greater peak activation of the EDL compared to the KT and the control group during first landing and less activation decay during take-off. Given the rigid and supportive nature of the RT bandage, it likely promotes greater muscle activation to compensate for the limitation imposed by the material. This could be especially relevant in the take-off phase, where substantial muscle activation is required to generate force and propel the body upward in the vertical jump. Furthermore, the evertor component of the EDL muscle could stop the typical inversion injury mechanism that leads to ankle sprains [64].

The increased EDL activation observed in the RT condition suggests a distinct neuromuscular control strategy compared to KT. While KT appears to facilitate pre-activation mechanisms through sensory feedback, RT may enforce a compensatory response where greater muscle activation is required to overcome external restriction. This could imply that RT promotes an alternative stabilization strategy relying more on reactive muscle activation rather than preparatory adjustments. Future research should evaluate whether these differences translate into distinct injury prevention or performance enhancement effects in dynamic sports settings.

Our findings differ somewhat from previous research. For instance, Karlsson et al. [65] reported that taping an unstable ankle increased PL activation, reducing response time. In contrast, our study showed greater activation in the EDL. Although both muscles have an abduction and pronation component, they differ in their action in the sagittal plane: the peroneal muscles perform plantar flexion while the EDL performs dorsiflexion. This discrepancy could be explained by differences in biomechanics and muscle function between subjects with ankle instability and healthy subjects. In an unstable ankle, the tape can influence the peroneal muscles to stabilize the joint. However, in healthy individuals, the RT could increase activation in a pure evertor muscle, which could be a more effective strategy to compensate for inversion movements. On the other hand, subjects with instability may have greater difficulty stabilizing the ankle joint during such movements. In summary, the discrepancy between our findings and previous studies could be related to differences in the population studied and the specific muscle actions involved in ankle stabilization.

Consistent with our results, Yoon et al. ([66]) investigated the effect of RT on muscle activation of the TA and GM, without observing significant differences in the EMG activity during the heel-off and toe-off phases during inclined plane walking. However, between the heel strike and heel-off phases of the gait cycle, the mean EMG activity of the GM muscle increased significantly, and the activity TA activity decreased. It is important to consider the difference in the selected sample, since Yoon’s study used a sample of young women with limited ankle dorsiflexion, in contrast to our study’s active and healthy individuals. Such variations in sample characteristics—including age, activity level, and anatomical limitations—can considerably influence muscle response to taping interventions [67]. Similarly, Yin et al. ([68] did not find significant differences in PL and GM muscle activation comparing tape and KT in individuals with chronic ankle instability. However, a significant reduction in muscle activation of both muscles was observed with the tape application compared to the absence of taping during different types of perturbations and sensory stimuli.

Taken together, these findings highlight the importance of understanding how KT and RT influence neuromuscular control through different mechanisms. KT appears to enhance proprioceptive feedback and pre-activation strategies, whereas RT may rely more on reactive stabilization and increased muscle activation. Future studies should incorporate motion analysis and postural control assessments to better characterize these mechanisms and their implications for injury prevention and performance optimization.

The discrepancies in EMG findings may also be due to differences in joint speeds and loads across various studies. In our study, by using a fatigue protocol involving treadmill running and a SLDJ, we replicate the dynamic conditions found in continuous running, addressing the questions and limitations raised by other studies [69]. The SLDJ is particularly relevant as it mimics the repetitive single-leg stance and landing actions observed in running, where stability and muscle activation are crucial for performance and injury prevention. These activities involve higher joint speeds and loads compared to the static or low-intensity assessments of previous research, providing a more realistic context for evaluating muscle activation and stability under fatigue. This approach enhances the applicability of our findings to practical sports settings, contributing to the understanding of how different taping methods affect muscle function under fatigue conditions. Additionally, our results offer insights for future research on injury prevention strategies and for clinical practices focused on optimizing taping techniques for athletes.

### Limitations, Clinical Implications, and Future Lines of Research

The present study has a series of limitations that must be considered. By following the standard guidelines for the application of the electrodes according to the SENIAM guidelines, it is possible that the time in which the subject completes the fatigue protocol, the skin is prepared, the electrodes are placed, and the test is performed could have influenced the observation of fatigue. However, the process was carried out as quickly as possible while ensuring proper skin preparation, which was necessary to guarantee optimal electrode adhesion. Additionally, the equipment was pre-prepared and calibrated to minimize delays.

In addition, the fatigue protocol may not have specifically affected the muscles tested, with cardiovascular fatigue being more pronounced, but cardiovascular fatigue can also affect muscle performance by limiting oxygen and nutrient supply. In this context, it is essential to consider both central and peripheral fatigue. Central fatigue, which originates in the central nervous system, leads to reduced activation of motor units and decreased excitability of motor neurons. Peripheral fatigue, which occurs at the muscle level, is characterized by metabolic changes that affect contractile efficiency and reduce the speed of muscle contraction. Both forms of fatigue affect neuromuscular function, contributing to decreased performance.

However, the aim was to mimic real-life running conditions, where it is a well-known fact that cardiovascular fatigue occurs in continuous running, reflecting the challenges athletes face during prolonged efforts.

Nevertheless, a potential limitation of the study design is the lack of a control group performing the same tests without fatigue induction. This would allow a better understanding of the specific effects of fatigue on muscle response and taping effectiveness. Future studies should incorporate such a control condition to isolate the direct impact of fatigue.

However, it would be interesting to conduct more studies using a specific Delayed Onset Muscle Soreness (DOMS) protocol for the ankle muscles to assume total muscular fatigue. Furthermore, the homogeneity of the sample and the cross-over study design could have contributed to the lack of observed differences. The participants included in the study were well-trained (averaging 27.3 km per week), suggesting that they may have had similar levels of fitness, sporting experience, and pure, automatic patterns of taking off, jumping, and landing. This phenomenon could have reduced the variability in muscle response between the different taping conditions.

To mitigate this limitation in future research, a more heterogeneous sample, including athletes of varying training levels and movement patterns, could provide greater variability in muscle responses. Additionally, incorporating a parallel-group study design, rather than a cross-over approach, might help differentiate the effects of taping under different fatigue conditions.

Future studies could evaluate the influence of running tests on uneven terrain, which may produce even more fatigue, to gain a more comprehensive understanding of taping in these conditions. Variables such as surface instability, slope, changes in terrain texture and unpredictable terrain reactions may impose greater neuromuscular demands, requiring increased proprioceptive feedback, altered joint loading and increased muscle coordination. These factors may exacerbate central and peripheral fatigue, providing further insight into the effectiveness of different taping methods under more demanding real-world conditions.

Furthermore, future research should explore different fatigue induction protocols that better isolate muscle fatigue from cardiovascular fatigue. Implementing task-specific fatigue protocols, such as repeated plyometric exercises targeting the ankle muscles, could help determine whether the observed effects are primarily muscular or systemic.

## Conclusions

The RT bandage resulted in decreased ankle dorsiflexion ROM in comparison to both KT bandage and a control group during pre-fatigue protocol measurements in the lunge test and in the Y Balance Test, specifically in the anterior direction. This reduction in dorsiflexion could have significant implications for athletic performance, as limited ankle mobility is associated with an increased risk of injury, particularly in movements requiring high levels of flexibility and force absorption.

Concerning flight time and height in the SLDJ, the RT bandage proved beneficial in mitigating fatigue symptoms before and after the treadmill protocol, which is crucial to maintain performance and reduce the risk of injury during prolonged activities. Regarding the RT bandage’s specific effects on muscle activation, our findings indicate that the RT group exhibited higher peak activation of the EDL muscle compared to the KT and control groups during initial landing, with a lesser decay in activation during take-off. Therefore, the RT bandage may lead to increased activation in a pure evertor muscle, suggesting a potentially effective strategy for compensating inversion movements. On the other hand, the KT showed a significant increase in the activation of the PL muscle during the first contact phase of the SLDJ, which could represent a protective behavior and a preventive mechanism against ankle instability.

Future research could further explore these findings by testing different populations, additional tape types or evaluating the impact of tape wraps on other athletic activities or terrains to broaden the practical applications of these interventions.

## Supporting information

S1 File Study protocol, Spanish version. (DOCX)

S2 File Study protocol, English version. (DOCX)

S3 File Dataset. (XLSX)

S4 File Checklist. (DOCX)

## Abbreviations

KT: Kinesiotape
RT: Rigid Tape
ROM: Range of motion
EMG: Electromyographic
SLDJ: Single Leg Drop Jump
BMI: Body Mass Index
TA: Tibialis anterior
EDL: Extensor digitorum longus
PL: Peroneus longus
MG: Medial gastrocnemius
SENIAM: Surface Electromyography for the Non-Invasive Assessment of Muscles
MVIC: Maximum voluntary isometric contraction
RSI: Reactive Strength Index
SEBT: Modified Star Excursion Balance Test
SL-CMJ: Single-leg counter movement jump
DOMS: Delayed Onset Muscle Soreness
