# Supplementary material for: Effects of tape and Kinesiotape on ankle range of motion, Single Leg Drop Jump and balance after running-induced fatigue: a cross-over clinical trial
Source: PLoS One. 2025 Apr 21;20(4):e0320152. doi: 10.1371/journal.pone.0320152 (PMC12011296; doi:10.1371/journal.pone.0320152)
Supplement: S1 File — (DOCX) [file pone.0320152.s001.docx]

**PROTOCOLO DE ESTUDIO – ESTUDIO VENDAJES**

**Diseño del estudio y consideraciones éticas**

Se llevará a cabo un ensayo clínico longitudinal cruzado con tres brazos de intervención en el Laboratorio de Rendimiento Deportivo de la Universidad Europea de Madrid (España), siguiendo la Declaración de Helsinki. Cada participante completará y firmará un formulario de consentimiento por escrito antes de ser incluido en el estudio.

**Cálculo del tamaño de la muestra**

El tamaño de la muestra se calculará utilizando el software G*Power 3.1.9.2 (G*Power©, Universidad de Dusseldorf, Alemania). Este análisis empleará una hipótesis de dos colas, una probabilidad de error alfa de 0,05 y un error beta de 0,2. Un estudio piloto que involucrará a 10 participantes analizará el tamaño del efecto eta cuadrado parcial para el resultado primario, que fue la altura en el SLDJ entre los grupos y el tiempo.

**Participantes**

Los criterios de inclusión serán (1) hombres y mujeres (2) entre 18 – 45 años (3) bien entrenados, con un programa de entrenamiento semanal de al menos dos días y un total de 20 km de carrera. Los criterios de exclusión vendrán determinados por la presencia de patología musculoesquelética de miembros inferiores o lumbopélvica en el último año.

**Aleatorización**

La aleatorización de los participantes se realizará con la función aleatoria de Microsoft Office Excel (Microsoft Corporation, Redmond, WA, USA).

**Variables**

**Variables sociodemográficas**

Se recopilarán como datos descriptivos el sexo (masculino o femenino), la edad (años), la altura (cm), el peso (kg), el índice de masa corporal (IMC) (kg/cm2), la extremidad dominante (derecha o izquierda) y el historial de esguinces de tobillo del deportista. Además, se recopilarán las siguientes características del entrenamiento semanal: número de días de entrenamiento, distancia recorrida (km), ritmo medio (min/km), presencia de fuerza muscular específica en miembros inferiores y core o entrenamiento propioceptivo.

**Estabilidad de las extremidades inferiores:** El Y balance test se utilizará para analizar la estabilidad del miembro inferior dominante. Los participantes se situarán en el centro de la zona en forma de Y. Mientras mantienen el equilibrio sobre una pierna, tendrán que alcanzar la mayor distancia posible con la otra extremidad sin caerse a lo largo de cada una de las tres ramas de la Y: anterior, posterior-derecha y posterior-izquierda. Las medidas se tomarán desde la punta del pie en cada dirección, y se realizarán tres ensayos para obtener un promedio.

**Movilidad en dorsiflexión del tobillo:** Se medirá a través de la aplicación MyROM durante la prueba de Lunge. En posición de caballo, con la extremidad dominante en el suelo y las manos en la cintura, se indicará a los participantes que se inclinen hacia adelante lo máximo posible sin despegar el talón. Se colocará un teléfono móvil a lo largo de la tibia para rastrear el ángulo de dorsiflexión y se registrarán las mediciones en la pantalla. Cada participante se someterá a tres mediciones y se calculará la media entre las mediciones.

**Activación electromiográfica de los músculos del tobillo:** Se medirá durante un Single Leg Drop Jump (SLDJ), en las siguientes fases: primer contacto, despegue y segundo contacto. Se evaluarán los valores de pico mínimo, promedio y pico máximo. Para evaluar la activación EMG, se evaluarán el tibial anterior (TA), el extensor largo de los dedos (EDL), el peroneo largo (PL) y el gastrocnemio medial (MG) utilizando el equipo de análisis EMG Delsys (Trigno Avanti; Natick, EE. UU.), evaluando el promedio total y los ratios de contracción.

Para la preparación de la piel y la aplicación y colocación de los electrodos se seguirán las pautas descritas en la Electromiografía de Superficie para la Evaluación No Invasiva de Músculos (SENIAM). (1) TA: El electrodo se coloca en la parte delantera de la pierna, sobre el vientre muscular. El punto medio es aproximadamente un tercio de la distancia entre la tibia y el borde externo. (2) EDL: El electrodo también se coloca en la parte delantera de la pierna, sobre el vientre muscular. El punto medio del músculo es un tercio de la distancia entre la tibia y el borde externo. (3) PL: El electrodo se sitúa en la región lateral de la pierna, sobre el vientre muscular. El punto medio se encuentra a un tercio de la distancia entre la tibia y el borde externo. (4) MG: El electrodo se coloca en la parte trasera de la pierna, sobre el vientre muscular. El punto medio es aproximadamente un tercio de la distancia entre la parte inferior de la pantorrilla y la parte superior del talón.

Para normalizar la señal EMG, se realizará una normalización basada en la activación durante la contracción isométrica voluntaria máxima (MVIC) de cada músculo. Estas evaluaciones se realizarán el primer día del protocolo de carrera (día 2). Los participantes ejecutarán tres contracciones isométricas contra resistencia durante 5 segundos, con 15 segundos de descanso entre repeticiones. Para el TA, los participantes estarán en posición sentada y realizarán dorsiflexión, aducción y supinación. Para el EDL, los participantes, en posición sentada, ejecutarán eversión pura. El PL se evaluará con los participantes en posición sentada, realizando flexión plantar, abducción y pronación. La evaluación MVIC del MG implicará que los participantes se acuesten boca abajo con la rodilla extendida, realizando inversión.

**Capacidad de saltar:** Se evaluará mediante el test SLDJ y se analizarán las características del salto con la aplicación MyJump 2. Esta prueba consiste en que el participante se coloca de pie sobre una pierna en una plataforma elevada y luego se deja caer rápidamente y salta inmediatamente en vertical con la misma pierna. La aplicación MyJump 2 permite recopilar varias variables clave, entre ellas:

- Altura del salto: Mide el desplazamiento vertical desde el salto inicial.
- Tiempo de vuelo: La duración que el participante pasa en el aire durante el salto.
- Tiempo de contacto: El tiempo que el pie del participante está en contacto con el suelo durante el salto.
- Índice de fuerza reactiva (RSI): se calcula como la relación entre la altura del salto y el tiempo de contacto, y proporciona información sobre la eficiencia del salto.

**Protocolo de intervención**

Los participantes voluntarios serán evaluados para determinar su elegibilidad, se recopilarán datos demográficos y se completará una prueba de carrera de esfuerzo máximo de 5 minutos en una pista de 400 m para determinar su velocidad aeróbica máxima individual.

Posteriormente, los participantes serán programados en tres sesiones (una vez por semana) para someterse a la intervención en tres condiciones diferentes, con el orden aleatorio: una sin vendaje (CONTROL), otra con vendaje de cinta, y otro con KT.

El vendaje será aplicado de forma consistente en la pierna dominante por un fisioterapeuta con diez años de experiencia clínica especializado en lesiones de tobillo y pie en corredores, dentro del Laboratorio de Rendimiento Deportivo de la Universidad Europea de Madrid.

Se realizará un calentamiento de 10 minutos basado en ejercicios de movilidad de miembros inferiores, estiramientos balísticos y carrera. Posteriormente se realizará una evaluación de la estabilidad de miembros inferiores, movilidad de dorsiflexión de tobillo, activación electromiográfica de la musculatura del tobillo y capacidad de salto, antes de iniciar el protocolo de cinta de correr.

Tras esta evaluación, se aplicará un protocolo de fatiga consistente en correr durante 30 minutos al 85% de la velocidad aeróbica máxima en una cinta. Una vez finalizado, se realizará una reevaluación inmediata de todas las pruebas administradas anteriormente, para recoger las mismas variables que al inicio del protocolo de carrera.

**Técnicas de vendaje**

***Vendaje KT***

En la técnica KT se emplearán tres segmentos de KT elástico: dos segmentos en configuración Y y un segmento en configuración I. Inicialmente, el segmento en configuración I se fijará longitudinalmente desde el maléolo tibial hasta el maléolo peroneo, recorriendo la porción anterior del tobillo. Posteriormente, se colocará el segmento alargado en configuración Y comenzando desde la faceta lateral del calcáneo hasta la cabeza del peroné, envolviendo el músculo PL. A continuación, se adherirá otro segmento más corto en configuración Y desde la faceta posterior del calcáneo hasta la base de la primera articulación metatarsofalángica, asegurando una cobertura integral del área anatómica objetivo.

***Vendaje de cinta***

El procedimiento de vendaje del tobillo se ejecutará utilizando una cinta autoadhesiva convencional de 38 mm, comenzando con la aplicación de dos correas de anclaje en una posición aproximadamente a 10 cm proximal a los maléolos. A esto le seguirá la colocación estratégica de dos correas adicionales que se extenderán desde el borde medial de la correa de anclaje hasta el lado lateral, con el pie mantenido en una posición neutra. Posteriormente, se elaborarán configuraciones en "figura de seis", comenzando con una tira que se origina en el anclaje medial, pasa por el aspecto plantar del pie y se vuelve a unir al anclaje medial. Para finalizar el procedimiento de vendaje del tobillo, el médico cubrirá meticulosamente todos los extremos terminales y cualquier laxitud con cinta adhesiva, asegurando una aplicación segura y uniforme.

**Cegamiento**

Debido a la naturaleza de la intervención, no habrá cegamiento de los participantes ni del fisioterapeuta que realizó el vendaje.

**Análisis estadístico**

El análisis estadístico se realizará con el programa SPPS versión 29 para Windows. En primer lugar, se evaluará la distribución de los datos con la prueba de Shapiro-Wilk o la prueba de Kolmogorov-Smirnov. A continuación, se realizará un ANOVA de una vía para analizar la diferencia entre los grupos al inicio del estudio. Finalmente, se realizará un ANOVA de dos vías de medidas repetidas (3 x 2; 3 grupos y 2 tiempos de medición) para analizar la diferencia entre grupos prey protocolo posterior a la cinta de correr. El investigador que realice el análisis estadístico será ciego. Todas las variables se analizarán individualmente y las comparaciones múltiples se tendrán en cuenta mediante una corrección de Bonferroni. El nivel de significación se establecerá en 0,05.
