# Supplementary material for: Effects of tape and Kinesiotape on ankle range of motion, Single Leg Drop Jump and balance after running-induced fatigue: a cross-over clinical trial
Source: PLoS One. 2025 Apr 21;20(4):e0320152. doi: 10.1371/journal.pone.0320152 (PMC12011296; doi:10.1371/journal.pone.0320152)
Supplement: S2 File — (DOCX) [file pone.0320152.s002.docx]

**STUDY PROTOCOL – TAPE TRIAL**

**Study design and ethical considerations**

A cross-over, longitudinal, clinical trial with three intervention arms will be conducted in the Sports Performance Laboratory of the European University of Madrid (Spain), adhering to the Helsinki Declaration. Each participant will complete and signed a written consent form before being enrolled in the study.

**Sample size calculation**

The sample size will be calculated using the G*Power 3.1.9.2 software (G*Power©, University of Dusseldorf, Germany). This analysis will employ a two-tailed hypothesis, an alpha error probability of 0.05, and a beta error of 0.2. A pilot study involving 10 participants will analyze the partial eta squared effect size for the primary outcome, which was the height in the SLDJ between groups and time.

**Participants**

The inclusion criteria will be (1) males and females (2) between 18 – 45 years old (3) well-trained, with a weekly training schedule of at least two days and a total of 20 km of running. The exclusion criteria will be determined by the presence of musculoskeletal lower limb or lumbopelvic pathology in the last year.

**Randomization**

The randomization of the participants will be carried out with the random function of Microsoft Office Excel (Microsoft Corporation, Redmond,WA, USA).

**Variables**

**Sociodemographic variables**

Athlete’s gender (male or female), age (years), height (cm), weight (kg), Body Mass Index (BMI) (kg/cm2), dominant limb (right or left), and history of ankle sprains will be collected as descriptive data. In addition, the following weekly training characteristics will be collected: number of training days, running distance (km), average pace (min/km), presence of specific lower limb and core muscle strength, or proprioceptive training.

**Lower limb stability:** The Y balance test will be used to analyze the stability of the dominant lower limb. Participants will stand in the center of the Y-shaped area. While balancing on one leg, they will have to reach as far as possible with the other limb without falling along each of the three branches of the Y: anterior, posterior-right and posterior-left. Measurements will be taken from the tip of the foot in each direction, with three trials conducted to obtain an average.

**Ankle dorsiflexion mobility** will be measured through the MyROM app during the Lunge test. In a knight's stance, with the dominant limb on the ground and hands on the waist, participants will be instructed to lean forward as far as possible without taking off the heel. A mobile phone will be placed along the tibia to track the dorsiflexion angle, and measurements will be recorded on the screen. Each participant will undergo three measurements and the mean between measurements will be calculated.

**Electromyographic activation of the ankle muscles** will be measured during the SLDJ, in the following phases: first contact, takeoff, and second contact. The minimal peak, average, and maximal peak values will be evaluated. To assess EMG activation, the tibialis anterior (TA), extensor digitorum longus (EDL), peroneus longus (PL), and medial gastrocnemius (MG) will be evaluated using the EMG analysis equipment Delsys (Trigno Avanti; Natick, USA), assessing the total average and ratios of contraction.

For skin preparation and electrode application and placement, the guidelines outlined in the Surface Electromyography for the Non-Invasive Assessment of Muscles (SENIAM) will be followed. (1) TA: The electrode is positioned on the front of the leg, over the muscle belly. The midpoint is approximately one-third of the distance between the tibia and the outer edge. (2) EDL: The electrode is also placed at the front of the leg, over the muscle belly. The midpoint of the muscle is one-third of the distance between the tibia and the outer edge. (3) PL: The electrode is situated in the lateral region of the leg, on the muscle belly. The midpoint is located at one-third of the distance between the tibia and the external border. (4) MG: The electrode is positioned on the back of the leg, over the muscle belly. The midpoint is approximately one-third of the distance between the bottom of the calf and the top of the heel.

To normalize the EMG signal, a normalization based on the activation during the maximum voluntary isometric contraction (MVIC) of each muscle will be performed. These evaluations will be conducted on the first day of the running protocol (Day 2). Participants will execute three isometric contractions against resistance for 5 seconds, with 15 seconds of rest between repetitions. For the TA, participants will be in a seated position and will perform dorsiflexion, adduction, and supination. For the EDL, participants, in a seated position, will execute pure eversion. The PL will be assessed with participants in a seated position, performing plantarflexion, abduction, and pronation. The MVIC assessment of the MG will involve participants lying prone with the knee extended, performing inversion.

**Jumping ability** will be assessed using the SLDJ test, and the characteristics of the jump will be analyzed with the MyJump 2 app. This test consists of a participant standing on one leg on a raised platform and then quickly dropping down and immediately jumping vertically with the same leg. The MyJump 2 app allows for the collection of several key variables, including:

- Jump Height: Measuring the vertical displacement from the initial jump.
- Flight Time: The duration the participant spends in the air during the jump.
- Contact Time: The time the participant's foot is in contact with the ground during the jump.
- Reactive Strength Index (RSI): Calculated as the ratio of jump height to contact time, providing insights into the efficiency of the jump.

**Intervention protocol**

Voluntary participants will be assessed for eligibility, collection of demographic data, and completion of a maximal effort 5-minute run test on a 400-m track to determine their individual maximal aerobic speed.

Subsequently, participants will be scheduled for three sessions (once a week) to undergo the intervention under three different conditions, with the order randomized: one without a bandage (CONTROL), one with a tape bandage, and another with KT.

The bandaging will be consistently applied to the dominant leg by a physiotherapist with ten years of clinical experience specializing in ankle and foot injuries in runners, within the Sports Performance Laboratory of the European University of Madrid.

A 10-minute warm-up will be performed based on lower limb mobility exercises, ballistic stretching and running. Subsequently, an assessment of lower limb stability, ankle dorsiflexion mobility, electromyographic activation of the ankle muscles, and jumping ability will be performed, before starting the treadmill protocol.

Following this evaluation, a fatigue protocol will be implemented, involving a 30-minute run at 85% of the maximal aerobic speed on a treadmill. Once completed, an immediate reevaluation will be conducted for all previously administered tests, to collect the same variables as at the beginning of the running protocol.

**Taping techniques**

***KT Bandage***

In the KT technique, three segments of elastic KT will be employed: two segments fashioned into Y-configurations and one segment in an I-configuration. Initially, the I-configured segment will be affixed longitudinally from the tibial malleolus to the peroneal malleolus, traversing the anterior portion of the ankle. Subsequently, the elongated Y-configured segment will be placed commencing from the lateral facet of the calcaneus extending to the fibular head, enveloping the PL muscle. Thereafter, another shorter Y-configured segment will adhere from the posterior facet of the calcaneus to the base of the first metatarsophalangeal joint, ensuring comprehensive coverage of the targeted anatomical area.

***Tape Bandage***

The ankle taping procedure will be executed utilizing a conventional 38 mm self-adhesive tape, initiating with the application of two anchor straps at a position approximately 10 cm proximal to the malleoli. This will be followed by the strategic placement of two additional straps extending from the medial edge of the anchor strap to the lateral side, with the foot maintained in a neutral position. Subsequently, "figure six" configurations will be crafted, commencing with a strip originating from the medial anchor, passing through the plantar aspect of the foot, and reattaching to the medial anchor. To finalize the taping ankle procedure, the practitioner meticulously will cover all terminal ends and any laxity with adhesive tape, ensuring a secure and uniform application.

**Blinding**

Because of the nature of the intervention, there will be no blinding of the participants or the physiotherapist who performed the taping.

**Statistical analysis**

The statistical analysis will be performed with the SPPS software version 29 for Windows. First, data distribution will be assessed with the Shapiro-Wilk test or Kolmogorov-Smirnov test. Then, a one-way ANOVA will be performed to analyze the difference between groups at baseline. Finally, a two-way ANOVA of repeated measures (3 x 2; 3 groups and 2 times of measurement) will be carried out to analyze the difference between groups pre and post treadmill protocol. The investigator who performs the statistical analysis will be blinded. All variables will be analyzed individually, and multiple comparisons will account for using a Bonferroni correction. The significance level will be set at 0.05.
